# A Case Report on a Vascular Malformation in the Abdominal Cavity

**DOI:** 10.7759/cureus.81366

**Published:** 2025-03-28

**Authors:** Nathaniel Z Wang, Nathaniel G Blanchard, Abby L Cummings, Paul Kowalski, Joyce DeJong, William McMillan, Nicole L Geske, Libby J Bradley

**Affiliations:** 1 Radiology, College of Osteopathic Medicine, Michigan State University, East Lansing, USA; 2 Radiology, Michigan State University, East Lansing, USA; 3 Pathology, Michigan Pathology Specialists PC - Spectrum Health, Grand Rapids, USA

**Keywords:** adhesions, anatomy, bowel histology, elastin, histology and histopathology, prosection, sigmoid colon

## Abstract

This case study presents the routine prosection findings of a 93-year-old male anatomical donor. A detailed examination of the abdomen revealed a fibrotic-like tissue stemming from the sigmoid colon to the peritoneum of the lower left anterior abdominal wall, 4 cm inferior to the left arcuate line. In total, the anomalous tissue measured 12 cm in length. Other noticeable pathological features included diverticulosis of the sigmoid colon and a left inguinal hernia. Gross anatomical and histopathological findings suggest that the tissue is a vascular malformation (VM). It is imperative to share our comprehensive findings with the medical community so that collaborations between surgeons and pathologists can provide optimal care for patients with similar anatomical findings.

## Introduction

Vascular malformations (VMs) are abnormal congenital or acquired conditions commonly defined by vessel abnormalities within channels rather than a mass [1,2]. These can vary, including lymphatic, venous, arterial, or capillary vessels. Some malformations can be a combination of vessel types. A single layer of endothelial cells lining the channel encasing the vessel(s) is the identifying feature of VMs [2]. Congenital VMs can be attributed to unsuccessful vasculogenesis as an embryo in the stage of capillary plexus formation, which occurs in the third week of embryonic development [2]. Mutations involving endothelial cell receptors and/or smooth muscle phenotype in the process of vasculogenesis generate the formation of these vascular abnormalities [1]. VMs, when congenital, will grow with the individual. However, acquired malformations can present later in life following trauma or after an inciting event. Detecting VMs is not common until later in life when symptoms such as pain or swelling occur. Specifically, VMs are rare in the lower gastrointestinal (GI) tract. When encountered, they typically appear as flat or mildly elevated lesions in the mucosal lining. Even more uncommon are cases where VMs in the colon present as pedunculated or polypoidal lesions, with only a few reported instances in the literature [3-5].

There is no cure for VMs; however, embolization, sclerotherapy, or surgery are typical treatment options. The severity and flow type of the VM are analyzed to determine the best treatment option for the patient [1]. Both embolization and sclerotherapy require multiple repetitions, but they have been found successful in treating VMs [6].

## Case presentation

Pathoanatomical findings

**Figure 1:**
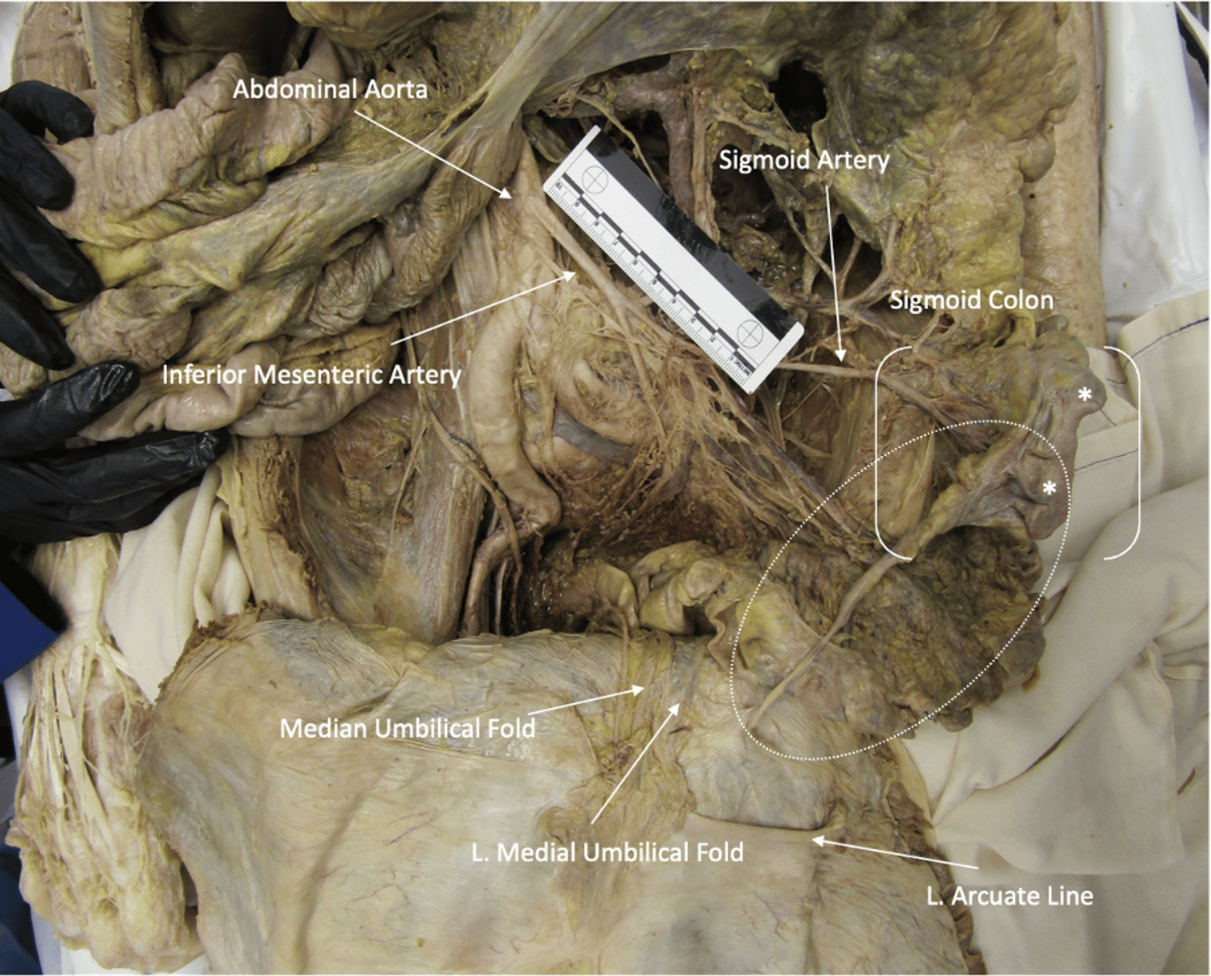
Abdominal anomaly in relation to prominent structures Anterior view of the abdomen, with the anterior abdominal wall reflected inferiorly. The tissue anomaly (outlined by an oval) measures approximately 12 cm in length and is attached to the sigmoid colon (in brackets) and the abdominal wall. Outpouching of the sigmoid colon (indicated by *) suggests diverticulosis.

**Figure 2:**
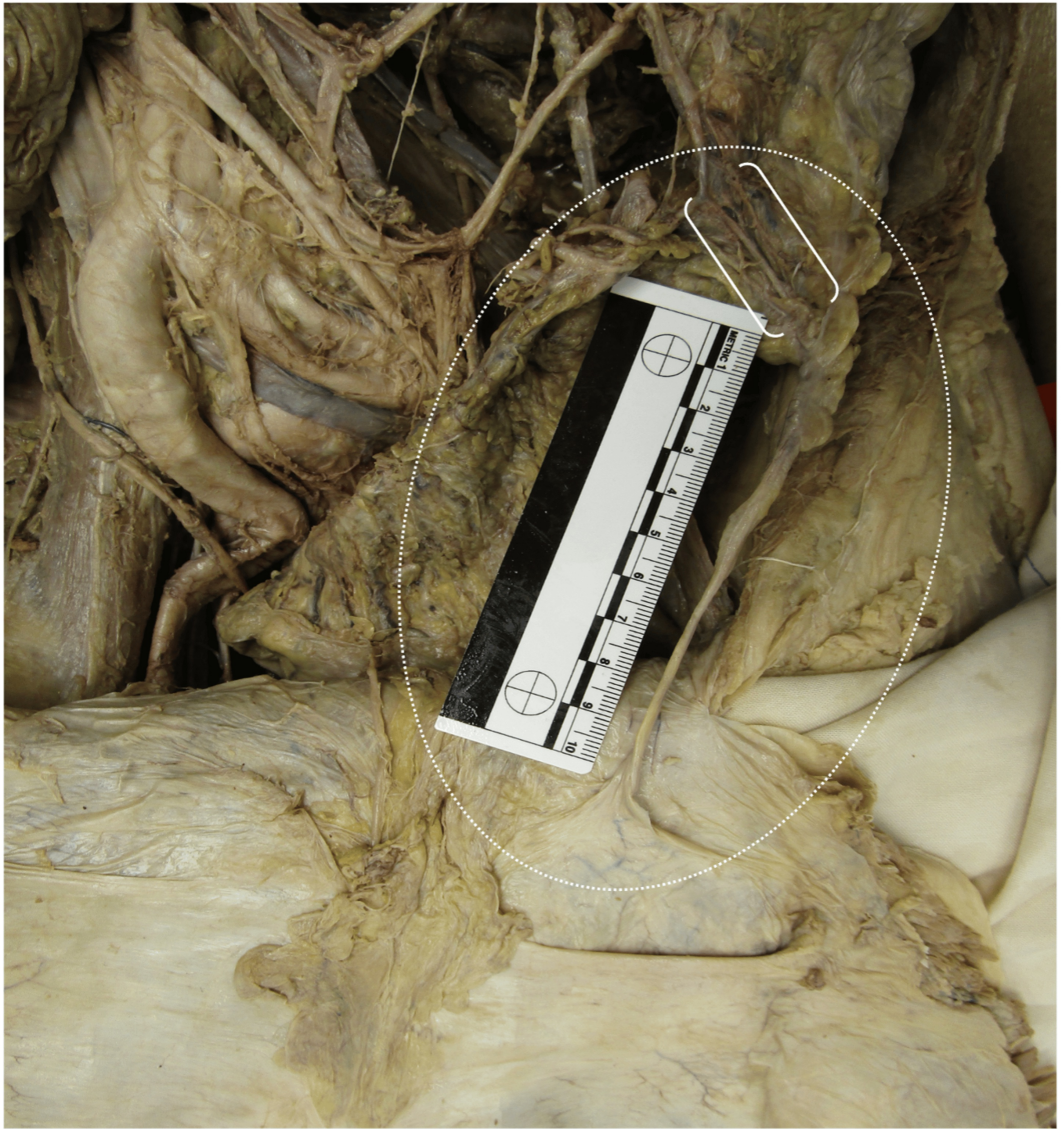
Vascularization of abdominal anomaly Highlighting the varying widths and lengths throughout the tissue anomaly (outlined by an oval). The tissue is evidently cylindrical with the thinnest and thickest sections ranging from 2-3 mm to 6-7 mm in diameter, respectively.  The length is approximately 12 cm. In addition, an arterial anastomosis between the left colic and sigmoidal arteries (outlined by brackets) traverses into the abdominal tissue anomaly.

During a routine prosection of a 93-year-old male anatomical (cadaveric) donor (“donor”), an apparent and significant fibrotic-like tissue was discovered. The tissue spanned from the sigmoid colon to the peritoneum of the lower left anterior abdominal wall, 4 cm inferior to the left arcuate line (Figure 1). The tissue was cylindrical in shape, with the thickest section measuring approximately 6-7 mm in diameter near the sigmoid colon. It gradually decreased in diameter to approximately 2-3 mm when reaching the anterior abdominal wall (Figure 2). The total length of the fibrous strand was 12 cm (Figure 2). The sigmoid mesocolon appeared to envelop the tissue, and several visceral fat particles approximately 2 cm in thickness were attached to the fibrotic tissue. 

In addition, there was overt vascularization of the tissue as demonstrated by an arterial anastomosis between the left colic artery and the sigmoidal arteries. This anastomosis also traversed into the thickest part of the cylindrical tissue near the sigmoid colon (Figure 2). The left colic and sigmoidal arteries otherwise demonstrated their typical branching patterns, and the corresponding left colic and sigmoidal veins appeared to provide a venous return from the anomalous tissue, following a similar pathway as the arteries (Figure 5 in Appendices). The cause of death of the donor was listed as acute on chronic diastolic heart failure. Prior to death, the patient had an extensive list of comorbidities, which included malnutrition, hypothyroidism, a cerebral vascular accident, an unspecified seizure disorder, and melanoma. During prosection, two additional anatomical pathologies were noted, including diverticulosis in the sigmoid colon and a left inguinal hernia (Figure 1). 

Histopathological findings

**Figure 3:**
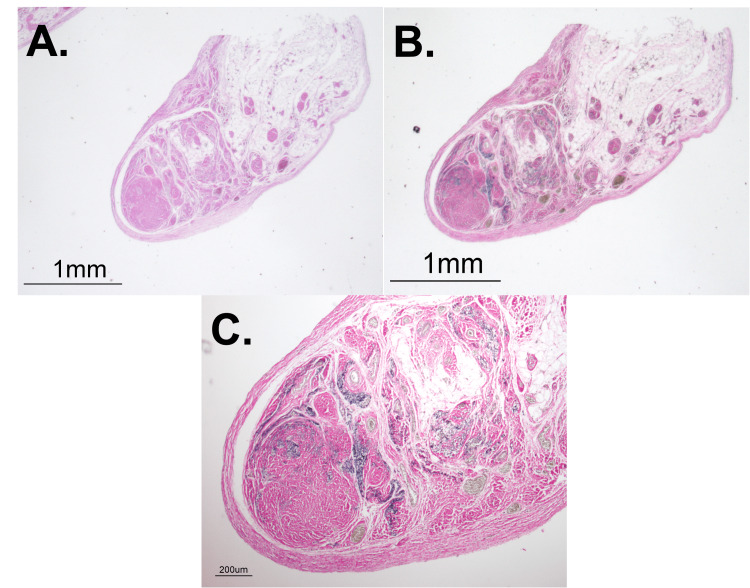
Cross-sectional staining with H&E and VVG A: Cross-sectional H&E stain at 1.25X. A significant number of adipocytes, with evidence of fat necrosis and neutrophil infiltration. Thick smooth muscle surrounds an abundant number of vascular structures. B: Cross-sectional VVG stain at 1.25X. Diffuse and haphazard elastin border surrounding vascular tissue and leaking into smooth muscle cells. C: Cross-sectional VVG stain at 4X. Abnormally thick and widely dispersed internal elastic lamina. H&E, Hematoxylin and Eosin; VVG, Verhoeff-van Gieson

Since the context surrounding the anatomical anomaly (e.g., the shape, location, and texture) did not provide an obvious identification and etiology, the authors had the sample histologically investigated. Cross-sectional samples were taken along the elongated mass spanning from the sigmoid colon to the abdominal wall. The samples were stained with routine Hematoxylin and Eosin (H&E) and Verhoeff-van Gieson (VVG) stains. H&E staining revealed that the cross-sectional samples showed the anomaly to have a substantial fibrous border, providing gross structural integrity (Figure 3A). Additionally, within the fibrous capsule, a significant number of adipocytes were present, as shown in Figure 3A. There was also evidence of fat necrosis with neutrophil infiltration among some adipocytes. More notably, however, thick smooth muscle surrounded a large number of vascular structures (Figure 3A). The prominence of the smooth muscle throughout the elongated mass accounted for the gross firmness of the anatomical structure.

**Figure 4:**
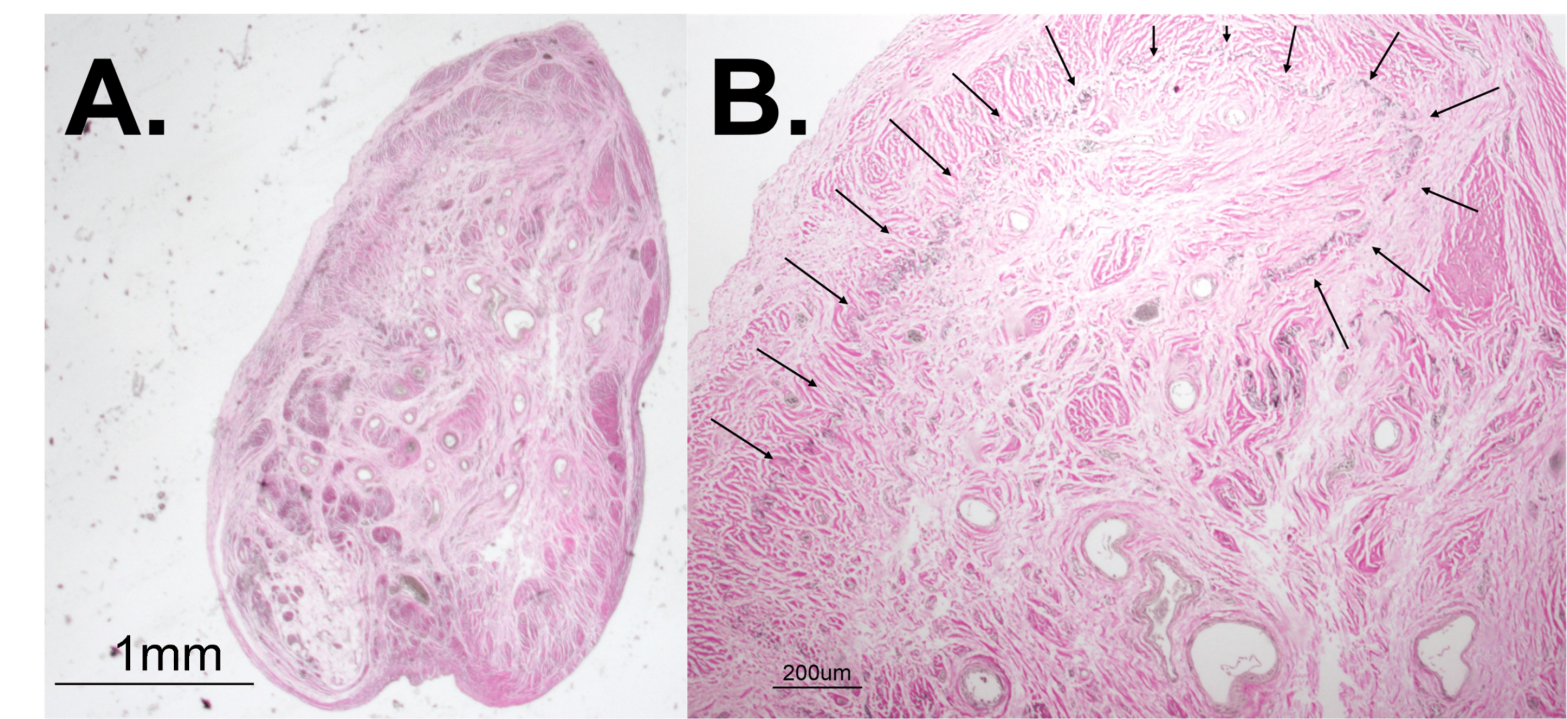
Prominent elastin border with VVG stain A: Cross-sectional VVG stain at 1.25X. The outline of a stained elastin border can be seen near the top of the cross-section. B: At a closer (4X) view, a prominent elastin border (arrows) can be seen in smooth muscle. VVG, Verhoeff-van Gieson

**Figure 5:**
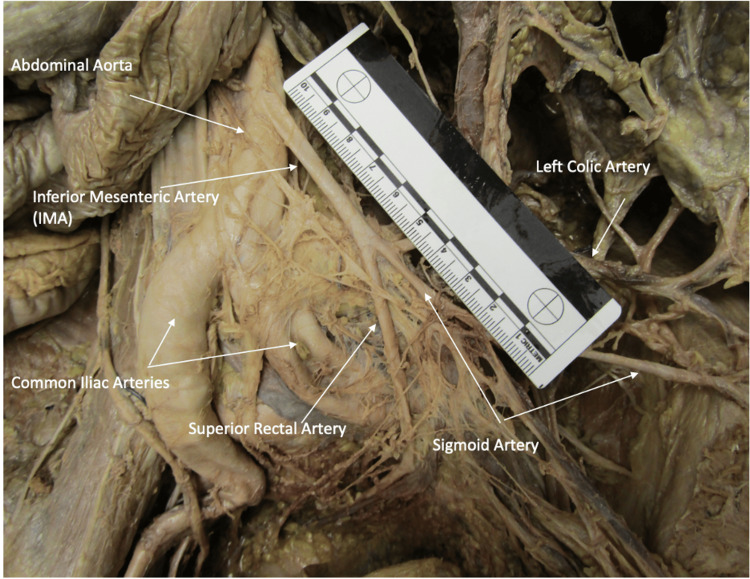
Vasculature of the hindgut Anterior view of the hindgut vasculature. The inferior mesenteric artery branches off the abdominal aorta, which then branches into the left colic artery, sigmoidal arteries, and superior rectal artery.

Because of the authors’ suspicion of vascularization from gross observation, VVG staining was also used to further characterize the elastin within the vascular wall and surrounding tissue. Upon investigation, there appeared to be a diffuse and haphazard border of elastin that lazily surrounded the vascular tissue and leaked into the adjacent smooth muscle cells (Figure 3B). Normally, VVG should stain an internal elastic lamina that is prominent, relatively thin, and directly adjacent to the vascular endothelial cells. The internal elastic lamina in these vessels, however, was abnormally thick and widely dispersed (Figure 3C). Additionally, there appeared to be an elastin border within the smooth muscle that could be evidence of a past vessel obliterated and now replaced by smooth muscle (Figure 4A and Figure 4B). The histological findings were consistent for all cross-sections spanning the length of the entire specimen.

## Discussion

Based on the gross features observed in this abdominal specimen, as displayed in Table 1, and supported by the histological findings, it is most likely that this case represents a VM. There have been many documented cases of VMs presenting in the colon, and as such, VMs have increasingly been identified as a source of lower GI bleeding [4-6]. While it is not uncommon for VMs to present as flat or slightly elevated bright red lesions in the mucosal lining of the colon, VMs can also present on rare occasions as pedunculated and polypoidal in nature [4,6]. Considering that published case studies on VMs in the colon have documented variations in anatomical presentation compared to established gross norms, the authors contend that it is entirely plausible for a VM to manifest in the manner observed in the donor [5]. The proposed hypothesis is also reinforced by the presence of vascular structures, particularly venous, emerging from the fibrotic-like tissue near the sigmoid colon, which drained blood into the left colic and sigmoidal veins. This is consistent with the characteristics of VMs, which typically exhibit both arterial and venous components that ultimately integrate with normal vascular architecture [2]. 

**Table 1:** Summary of pathoanatomical and histopathological results

Summary of pathoanatomical and histopathological results	
Pathoanatomical	Histopathological
Dense, fibrotic strand (12 cm in length).	Thick, fibrous border surrounding the anomaly.
Attached proximally from the sigmoid colon to the anterior abdominal wall.	Significant adipocytes with apparent fat necrosis involving neutrophils
Received arterial blood from left colic and sigmoidal arteries and involved corresponding colic and sigmoidal veins.	Faint, but broad sweeping, elastin border within anomaly. Additional haphazard distribution of elastin surrounding smaller vessels within the anomaly.
	Prominent smooth muscle.

Histological analysis, displayed in Table 1, further substantiates the diagnosis of a VM. The irregular distribution and haphazard arrangement of vessels, both arterial and venous, are hallmark features of such anomalies [7]. H&E staining revealed vessels extending throughout the length of the strand, with certain cross-sectional areas identifying as many as 15 to 20 individual vessels. These vessels exhibited significant variation in luminal diameter and extravascular composition. As VMs develop, vessels commonly appear dilated [7]. Some vessels demonstrate thin layers of surrounding connective and smooth muscle tissue, while others display thicker and irregularly distributed tissues, a pattern frequently observed in veins associated with VMs, as noted by Fernandez-Flores et al. [7]. 

Another characteristic of VMs is the irregular composition of vascular walls, particularly regarding the elastic fibers constituting the internal elastic lamina. These fibers can change with age, becoming fragmented or thickened. VVG staining revealed widespread patterns of irregular elastin deposition, including significantly thicker internal elastic lamina surrounding the vessels, with irregularly distributed elastic fibers within the surrounding extravascular smooth muscle. Notably, one VVG slide displayed a larger elastin ring encompassing multiple smaller vessels. VMs are susceptible to thrombotic events due to their irregular distribution, which can lead to recanalization as a biologic means to restore blood flow [7]. This outer elastin ring observed in the specimen suggests the possible historical presence of a more substantial vascular anomaly that could have been obliterated, subsequently giving rise to smaller vessels and connective tissue infiltration. 

While the hypothesis of a VM appears to be the most plausible explanation, it is essential to consider alternative diagnoses for the observed anatomical finding. Several aspects of the patient’s medical history suggest the possibility that the fibrotic-like tissue may represent an adhesion. Adhesions are fibrous scar tissue bands that can form within the abdominopelvic cavity and connect two or more intraabdominal surfaces or organs [8]. This pathology results from the disruption of mesothelial cells that line the abdominopelvic cavity [9]. The patient’s history of malnutrition and the presence of diverticulosis throughout the left descending and sigmoid colon could contribute to localized inflammation in the colonic tissue [1]. The observed diverticulosis supports this inflammatory process. As such, it is reasonable to hypothesize that such inflammation could extend to involve the surrounding mesothelial cells that line the abdominopelvic cavity, potentially initiating the formation of adhesion between the outer layers of the colon and the abdominal wall [10]. 

However, there are key findings that refute the diagnosis of an adhesion. First, the gross appearance of the tissue deviated from the typical characteristics of adhesions, which are often thick, fibrous bands connecting two abdominal surfaces [7]. In contrast, the fibrotic band in this case was notably thin and narrow, measuring over 12 cm in length and extending from the sigmoid colon to the anterior abdominal wall. Second, the vascularity observed in this tissue was atypical for adhesions. Adhesions are generally composed of dysregulated scar tissue with minimal vascularization, yet this case exhibited substantial vascularity, with cross-sections displaying up to 15-20 distinct blood vessels. Finally, histological examination, particularly with VVG staining, revealed dysregulated laminae and a lack of substantial fibrotic tissue, which further argues against adhesion identification. The dense, firm texture observed in the tissue appears to result from the presence of smooth muscle rather than fibrotic scar tissue, which would be expected in the case of an adhesion. 

It is essential for surgeons and pathologists to have a comprehensive understanding of both the gross presentation and histologic features of VMs, such as the one described in this case. This knowledge is critical in selecting appropriate treatment strategies, which may include endovenous laser therapy, sclerotherapy, surgical excision, embolization, or medical management [11].

## Conclusions

During the routine dissection of a 93-year-old male anatomical donor, a unilateral abdominal fibrotic tissue anomaly was identified. This tissue spanned from the sigmoid colon to the peritoneum of the lower left anterior abdominal wall. Subsequent histological analysis suggests that the most likely diagnosis is a VM. VMs are characterized by the abnormal development of blood vessels, often exhibiting distinct, dysregulated histological patterns, which was observed in this case. 

This case study aims to contribute to the understanding of similar anatomical presentations by hypothesizing the most probable etiology, supported by histological findings and a thorough review of the existing literature. By disseminating these observations, this study seeks to enhance the knowledge base for surgeons and pathologists, fostering improved collaboration in future cases. The identification of VMs like the one discovered in the donor will hopefully streamline appropriate treatment strategies and limit the need for extensive surgery time. Ultimately, the goal is to aid in more informed surgical decision-making and optimize patient care in related clinical contexts. Additionally, future research could focus on gathering data from similar presentations to identify commonalities and patterns that may help explain the cause of these malformations.
